# Significance of Serum Cytokeratin-18 in Prediction of Hepatocellular Carcinoma in Chronic Hepatitis C Infected Egyptian Patients

**DOI:** 10.3889/oamjms.2015.021

**Published:** 2015-02-17

**Authors:** Wafaa El-Zefzafy, Hanan Eltokhy, Nagwa Abd El-Ghaffar Mohamed, Zakia Abu-Zahab

**Affiliations:** 1 *Faculty of Medicine for Girls Al-Azhar University, Tropical Medicine, Cairo, Egypt*; 2 *Faculty of Medicine for Girls Al-Azhar University, Internal Medicine, Cairo, Egypt*; 3 *Department of Clinical and Chemical Pathology, National Research Center, AL-Tahreer Street, AL-Dokki, Giza, Cairo 12622, Egypt*; 4 *Faculty of Medicine for Girls Al-Azhar University, Clinical Pathology, Cairo, Egypt*

**Keywords:** Cytokeratin-18, viral hepatitis, HCC, α-fetoprotein, tumor marker

## Abstract

**BACKGROUND:**

Hepatitis C virus is one of the most common etiologic agents of chronic liver diseases, including liver cirrhosis and hepatocellular carcinoma in which there is continuous inflammation and regeneration of hepatocytes. Cytokeratin-18 (CK18) has been suggested to play an important role in tumorigenesis of epithelial cancers.

**AIM:**

Estimation of CK18 serum levels in patients with chronic viral hepatitis C (CHCV) and hepatocellular carcinoma (HCC) and find the relationship between their levels, the severity of the disease and the development of HCC.

**METHODS:**

We measured serum levels of CK18 in 60 Egyptian patients (30 with CHCV & 30 with HCC) and 30 healthy controls.

**RESULTS:**

Patients with HCC had highly significant increased CK18 levels compared with CHCV patients, healthy controls. Cytokeratin-18 among the three classes of Child-Pugh classification show highly significant gradual increase from child A to C. Furthermore, In HCC there were positive correlations between CK18 and with RBCs, ESR, and AFP.

**CONCLUSION:**

CK18 is a sensitive indicator of the severity of liver disease. Patients with CHCV infection can be followed up by measurement of its serum level which can predict the development of HCC. The combination of AFP and CK18 increased the sensitivity of detection for HCC.

## Introduction

Hepatitis C is a significant public health problem in Egypt where the prevalence is estimated to be 14, 7% in general population. Hepatitis C virus prevalence is even higher among clinical populations and groups at risk of exposure to infection [1].

Hepatitis C virus is considered the most common etiology of chronic liver disease (CLD) in Egypt. The disease severity ranges from mild illness to cirrhosis and hepatocellular carcinoma (HCC) [2] which are the most common causes of death in patients with (CLD). Chronic liver injury of virtually any etiology triggers inflammatory and wound-healing responses that in the long run promote the development of hepatic fibrosis and HCC [3].

Hepatocellular carcinoma was listed as the third most lethal cancer type [4]. The symptoms and signs of HCC are vague and rarely appear until the cancer has spread throughout the liver, if liver cancer is diagnosed in its early stages, it can be treated by surgically removing part of the liver, by liver transplantation, or by a radiofrequency ablation [5].

Prognosis for patients with HCC depends on tumor stage, with curative therapies only available for patients detected at an early stage. Patients detected at an early stage can achieve 5-year survival rates of 70% with transplant or resection, whereas those with advanced HCC are only eligible for palliative treatments and have a median survival of less than one year [6].

The diagnosis of HCC without pathologic confirmation can be achieved by assessing the serum alpha-fetoprotein (AFP) level combined with imaging techniques, including ultrasonography, magnetic resonance imaging, and computerized tomography [7], but effective test strategies should be considered to improve the early diagnostic rate of HCC including the combined detection of several serum markers that can complete each other in order to improve the early diagnostic rate [8].

Cytokeratins are proteins of keratin-containing intermediate filaments found in the intracytoplasmic cytoskeleton of epithelial tissue. The cytokeratins can be divided into low versus high molecular weight based on their molecular weight. Expression of these cytokeratins is frequently organ or tissue specific. In the cytoplasm, the keratin filaments conform a complex network which extends from the surface of the nucleus to the cell membrane [9].

Keratin 18 is a type I cytokeratin. It is, together with its filament partner keratin 8, perhaps the most commonly found products of the intermediate filament gene family. They are expressed in single layer epithelial tissues of the body. Mutations in this gene have been linked to cryptogenic cirrhosis. Two transcript variants encoding the same protein have been found for this gene [10].

Cytokeratin 18 is also called tissue polypeptide specific antigen representing about 5% of total protein in the liver, exocrine pancreas, intestine and other epithelial tissues [11]. CK18 has been suggested to play an important role in tumorigenesis of epithelial cancers [12]. Also Christoph etal., 2009 [13] found that soluble CK18 fragments (M30, M65) are released from human cancer cells during cell death.

In the study of Bantel and coworkers, raised concentrations of CK18 fragments were evident in HCV patients with liver fibrosis even in the absence of elevated aminotransferase [14].

The aim of this study was to estimate of CK18 serum levels in patients with chronic viral hepatitis C (CHCV) and hepatocellular carcinoma (HCC) and to find the relationship between their levels, the severity of the disease and the development of HCC.

## Patients and Methods

This study was conducted on 60 adult patients: 30 patients with CHCV infection and 30 patients with HCC who presented to Tropical, Internal Medicine Department of Al-Zahraa University Hospital from March to November 2014. The study also included 30 healthy subjects who were clinically, laboratory and ultrasonographically free served as a control group.

### Inclusion criteria

Adult patients (>18 years), both sexes (males or females) and patients with CHCV infection (either compensated or decompensated) or HCC were included.

### Exclusion criteria

Patients with history or evidence of other malignancies, patients suffering from any other organ failure and other causes of cirrhosis e.g. alcohol were excluded.

### Subject groups and investigations

Group 1 (G1): Included 30 patients with chronic HCV infection either compensated (18) or decompensated (12) they were (14 males, 16 females), their ages ranged from 35-60 years old with mean of (47.9 ± 6.67).

Group 2(G2): Included 30 patients with HCC on top of chronic HCV infection (22 males, 8 females), their ages ranged from 45-62 years old with mean of (55.4 ± 5.85).

Group 3: Included 30 healthy subjects with no clinical, laboratory or ultrasonographic evidence of liver disease served as a control group (13 males, 17 females), their ages ranged from 25-47 years old with mean of (32.2 ± 7.01).

Patients groups were sub-classified according to the severity of liver disease using modified Child’s-Pugh classification system to assess CK18 serum levels in relation to the progression of liver disease.

All patients and controls were subjected to the following:

1. Full history and clinical examination.
2. Abdominal ultrasonography.
3. Abdomino-pelvic triphasic CT scan for suspected cases of HCC. Liver biopsy and histopathological examination was done when needed for patients with hepatic focal lesions not fulfilling imaging or AFP diagnostic criteria for HCC.
4. Laboratory investigations: Three ml of fasting (6-8 hours) of fasting venous blood samples was taken from each subject participating in the study and divided into three portions as follows:
  - First portion was collected into Na citrate -containing tube, and used for estimation of prothrombin time (PT) immediately on automated blood coagulation analyzer Sysmex CA1500 (Siemens AG, Erlangen, Germany) and for ESR estimation by Westergren´s method.
  - The second portion was collected into EDTA containing tube for CBC estimation using Coulter Counter T890 (Coulter LH 750 analyzer, Berlin, Germany)
  - The third portion was put in a plan tube, left to clot then centrifuged at 1600 rpm for 20 minutes and serum was separated and used for estimation of:
  - Liver and kidney function tests were done on Hitachi 911 auto-analyzer (Roche-Hitachi, Japan).
  - Hepatitis makers (HBsAg, HCV Ab) based on Enzyme Linked Immunosorbent Assay (ELISA) technique, by ADVANCED HBsAG ELISA Test Kit Catalogue number; ITP21201Rev.40202, and for HCVAb in serum: Detected by ELISA third generation, using kit from ORTHO. Catalogue number; 631300942.

- Serum Alpha fetoprotein (AFP) was detected by COBAS e411 chemiluminescence auto analyzer using Roche reagents (Roche Diagnostics GmbH, D-68289 Mannheim, Germany) (Qin et al., 2011) [15].

- Serum CK18 assay was performed using ELISA kit supplied from DRG International Inc. (841 Mountain Avenue, Springfield, New Jersey 07081, USA, Catalogue number EIA 2354. Briefly, standards, controls and samples react during incubation simultaneously with a solid phase monoclonal catcher antibody and the HRP-conjugated detector antibody (M3). After washing, the TMB substrate is added and after an incubation time the reaction is stopped and the absorbance at 450 nm is measured. The level of CK18 was calculated from standard curve corresponding to the measured optical density. The results were expressed as U/L. Minimum detectable limit of CK18 ranged from 30 - 1200 U/L. [16].

### Statistical analysis

Data was analyzed using Microsoft Excel 2007. Parametric data was expressed as mean ± SD and non-parametric data was expressed as number and percentage. Student’s t test was done to compare between groups. Pearson Correlation Coefficient was done to correlate between different parameters among groups. Analysis of Variance (ANOVA) test was used to estimate the difference between the means of more than two groups. *P value of > 0.05* considered non significant, *P value of ≤ 0.05* considered significant, *P value of < 0.001* was considered highly significant.

## Results

**Figure 1:**
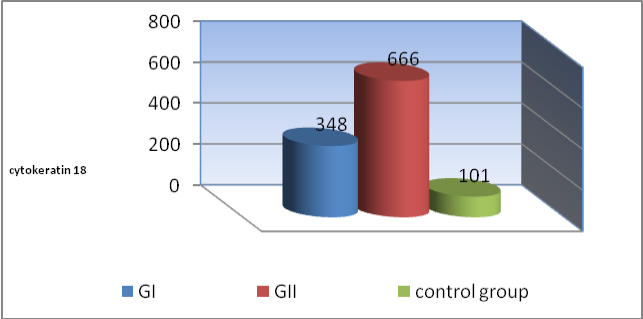
*Comparison of serum cytokeratin-18 among the studied groups*.

**Figure 2:**
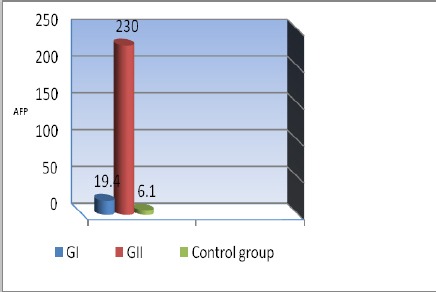
*Serum α-fetoprotein (AFP) among the studied groups*.

**Figure 3:**
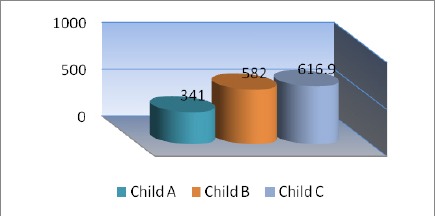
*Serum cytokeratin-18 among the three classes of Child-Pugh classification*.

**Figure 4:**
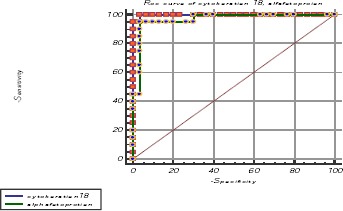
*The receiver operating characteristic curves cytokeratin-18, serum (AFP)*.

The results and data were collected and analyzed in tables 1-5 & Figures 1-4: As regard the result of personal data of the studied groups: there was highly significant difference in age among the studied groups with a significant male predominance in GII in comparison to GI (Table 1).

**Table 1:** Age and sex distribution of the studied groups.

	Groups		G 1 (n=30)	G 2 (n=30)	Control (n=30)	P-value
Age(years)	Mean ± SD		47.9 ± 6.67	55.4 ± 5.85	32.2 ± 7.01	0.001

Sex	Males	No.	14	22	13	0.03

		%	46%	73%	40%	

	Females	No.	16	8	17	

		%	54%	27%	60%	

G1: CHCV; G2: HCC.

**Table 2:** Comparison of complete blood picture among the studied groups (Mean ± *SD).*

	Groups			P		

	Group I	Group II	Control	GI/Control	GII/Control	G I/G II
WBCs (Thousands/cmm)	6.50 ± 2.98	6.6 ± 3.22	5.9 ±1.36	0.57	0.50	0.87

RBCs (Millions/cmm)	4.08± 0.46	4.5 ±0.4	4.23±0.72	0.35	0.76	0.43

Hemoglobin (gm/dl)	12± 2.20	10.8±1.73	12.8 ±1.94	0.39	0.010	0.062

Platelets (Thousands/cmm)	200± 93.7	116±50	255 ±39.8	0.086	<0.001	0.001

ESR (mm/H)	23.3±18.2	85.2 ±37.6	9.9±1.5	0.006	<0.001	<0.001

P>0.05: non significant; (P <0.05): significant; (p<0.001): highly significant.

**Table 3:** Comparison of Biochemical characteristics among the studied groups (Mean ±SD).

		G I (n=30)	G II (n=30)	Control (n=30)	P-value GI/Control	P-value GII/Control	P-value G I/G II
ALT IU/L	Mean	48.5	53.6	22.2	0.048	0.001	0.63

	±SD	39.6	25.6	5.57			

AST IU/L	Mean	54.5	57.7	25.1	0.017	0.01	0.78

	±SD	36.0	33.3	8.13			

PC%	Mean	65.6	53.3	96.4	<0.001	<0.001	0.029

	±SD	14.4	19.6	6.33			

Albumin gm/dl	Mean	3.24	2.56	4.31	<0.001	0.001	0.004

	±SD	0.803	0.564	0.303			

T.Bilirubing/dl	Mean	1.43	3.05	0.62	<0.001	0.004	0.01

	±SD	0.604	2.43	0.2			

Alkph IU/L	Mean	131	173	72.4	0.025	0.006	0.16

	±SD	76.6	106	18.4			

	±SD	102	95.6	26.0

**HS: highly significant at p<0.001; S: ≤ 0.05 significant; NS: non significant at p>0.05.

**Table 4:** Sensitivity, specificity, positive predictive value, negative predictive value, positive, negative (likelihood ratio) of cytokeratin-18, α-fetoprotein.

	Sensitivity	Specificity	PPV	NPV	+LR	-LR	Optimum cut-off point selected
CK18 (U/L)	95%	96.7%	94.7	96.7	28.4	0.054	543

AFP (ng/mL)	45%	96.6%	90	72.5	13.5	0.57	134

PPV (positive predictive value), NPV (negative predictive value), +LR (positive likelihood ratio), -LR (negative likelihood).

**Table 5:** Correlations between serum cytokeratin-18 serum levels and laboratory data in the studied patients (n=40).

	GI		GII	
	r	P-value	r	P-value
Age	0.319	0.175	0.006	0.977
Hemoglobin	-0.456	0.043	0.107	0.653
RBCs	0.09	0.705	0.126	0.652
WBcs	-0.303	0.194	0.012	0.959
Platelets	-0.679	<0.001	0.354	0.125
ESR	0.550	0.011	0.255	0.276
ALT	-0.048	0.840	-0.061	0.796
AST	0.160	0.499	-0.104	0.662
T.B	0.184	0.435	0.269	0.262
albumin	-0.555	0.011	-0.149	0.530
PC	-0.411	0.071	-0.376	0.102
Alkph	0.057	0.811	0.049	0.835
AFP	0.840	<0.001	0.996	<0.001

There were significant decrease in hemoglobin (Hb) level, in G II in comparison to GI, control group at P value (≤0.05). highly significant decrease in platelets count (PLT) count, increase in ESR in G II in comparison to GI &control group while no significant difference in WBCs, RBCs was detected among the studied groups (Table 2).

Our results revealed highly significant increase in ALT, decrease in prothrombin time (PT) & serum albumin in GII in comparison to control group at P value (<0.001) and significant increase in AST& Total(T)Bilirubin & alkalin phosphatase (ALKPH) in GI, GII in comparison to control group, with significant decrease in (PT) & serum albumin& T. Bilirubin in GII in comparison to GI (Table 3).

Concerning the result of serum AFP there was significant increase in its levels in GII in comparison to GI, control group with sensitivity and specificity 45% and 96.6%, respectively (3 patients in G2 with hepatic focal lesions, AFP was below diagnostic level for HCC so liver biopsy and histo-pathological examination was done) while serum CK18 was highly significantly increased in GII in com-parison to GI and control group with sensitivity and specificity of 95% and 96.7% (Fig. 1, 2), (Table 4).

CK18 among the three classes of Child-Pugh classification show highly significant gradual increase from child A to C in G1 and G2 patients (Fig. 3).

ROC Curve Analysis: Area under ROC curve at 95% CI for CK18 by extended trapezoidal rule = 0.998333 with Optimum cut-off point selected = 534.5 while for AFP it was = 0.966667 with Optimum cut-off point selected= 134 for HCC diagnosis (Fig. 4).

In GI there was significant negative correlation between serum CK18 & hemoglobin, platelets, serum albumin, positive correlation with RBCs, ESR, serum AFP while in GII there was only positive correlation with serum AFP (Table 5).

## Discussion

Hepatitis C virus (HCV) is a global health problem with an estimated 170-200 million peoples (approximately 3% of world population) are chronically infected worldwide and new infections are predicted to be on rise in coming years. HCV infection remains categorized as a major risk factor for chronic hepatitis, liver cirrhosis and hepatocellular carcinoma worldwide [17]. The discovery of an effective, reliable tool for early diagnosis of HCC to increase the number of patients who are suitable for curative treatment will play a pivotal role in improving HCC patients’ prognosis [7].

Liver cell damage in CHCV infection is mediated by the induction of apoptosis, the key morphological alterations of apoptosis are mediated by a family of intracellular cysteine proteases, called caspases (mainly caspase-3) [18], which cleave a number of different substrates inside the cell—including (CK-18), the major intermediate filament protein in the liver—resulting in the characteristic morphologic changes of apoptosis which has a role in liver damage caused by CHCV infection [19]. Studies have confirmed that CK-18 secretion occurs in parallel with DNA synthesis, protein synthesis, and cell division [20].

In the present study, the mean age of G 2 was higher than G 1, this result is similar to studies by Mittal, and El-Serag [21] as they found that in high incidence areas, HCC is reported to develop in the fifth decade of life. In our study, a male predominance was noted in HCC group, this was close to what was mentioned by Avunduk [22] who reported that HCC was more common in men with percentage of 80%.

As regard the blood picture, our study demonstrated highly significant decrease in platelets count in G 2 in comparison to GI and the control group. Lu etal., [23] reported that, thrombocytopenia was a valid surrogate of cirrhosis and a valid marker for the identification of individuals at high-risk for HCC, especially in areas that had a high prevalence of HCV.

No significant difference in total leukocytic count was observed in G 2 in comparison to G 1 or the control group, on the other hand Friedman [24] who categorized persistent leukocytosis as one of the paraneoplastic syndromes associated with HCC.

Our results revealed significant increase of serum ALT in GI in comparison to control group, significant increase of serum AST in GI, GII in comparison to control group. Bantel et al., [14] observed that more than 50% of patients with CHCV with normal aminotransferases exhibited already elevated serum caspase activity which revealed higher stages of fibrosis. Arturo etal., [25] reported that serum CK18 levels correlated with liver markers, particularly serum AST.

Moreover, in our study serum CK18, revealed a highly significant increase of its level in patients with CHCV this is in agreement with Arturo et al.,[25]. The mechanism causing increased CK18 concentrations in chronic hepatitis patients is unknown, but may be related to the marked sensitivities of this protein for nonneoplastic tissue in rapid regeneration, cell lysis, sinusoidal flow, and metabolism of glycoprotein markers [26]. On comparing the results of serum CK-18 in GI, GII, control group with that of aminotransferases we found that it may be a more sensitive marker for the detection of liver injury.

We demonstrated a highly significant difference in serum CK18 levels between patients with HCC and healthy controls. Our results agree with Dom-Gene et al., [27] however, they found the difference between the HCC and chronic hepatitis groups was not significant at (P = 0.18) but we found it highly significant at P <0.001. They added that a high serum Ck18 concentration would not be expected to distinguish HCC from hepatic metastases because this antigen is derived none selectively from malignant cells and elevated concentrations are found in the serum of patients with a wide variety of malignant diseases, some of which metastasize to the liver.

In our study the sensitivity and specificity of serum Ck-18 were 95% and 96.7%, respectively, with a cutoff value of 534.5 U/L for HCC diagnosis. While Dom-Gene et al., [27] found The sensitivity and specificity of serum Ck-18 were 73.1% and 71.2%, respectively, at a cut-off value of 164 (U/L) for a HCC diagnosis in Chinese patients.

Kao et al., [31] reported elevated serum Ck-18 levels in hepatitis B carriers with a low specificity for discriminating between hepatitis B carriers and HCC. In our study, we found progressive highly significant gradual increase in serum CK18 parallel to the severity of liver disease as assessed by Child’s-Pugh classification, these findings agree with the results of Collazos etal., [26].

Abdel Haleem et al., [32] found the serum level and the hepatic expression of CK-18 are related to disease activity and are directly correlated with METAVIR scoring So, monitoring the serum CK-18 levels can limit the need for liver biopsy for detection of their tissue levels.

In addition, serum CK18 levels correlated with Knodell’s score in patients with chronic hepatitis [25]. In contrast to our result no statistical correlation was found with liver markers except negative correlation with serum albumin in GI.

In GI& GII there was significant positive correlation between serum CK18 and serum AFP which has been regarded as the most useful serum protein thus far for patients at risk for HCC. However, its sensitivity for detecting HCC ranges between 25%-60% and its specificity is also low because serum AFP can also be detected in patients with cirrhosis (11%-47%) and chronic hepatitis (15%-58%) [4]. In addition to AFP, more than 20 serum proteins have clinical significance in early diagnosis of HCC [28], among which several proteins are proved to have advantages over AFP. There have been recent advances in technology that have enabled early identification of the process of hepatocarcinogenesis [29].

Concerning our result of (AFP) there was significant increase in its serum levels in HCC group in comparison to CHCV group, control group with no significant difference in CHCV group in comparison to control group.

We found serum concentrations of AFP elevated in 30% of CHCV patients with a mean value 19.4ng/mL. These results go parallel with the results of El-Serag et al., [4] while the percentage jumps to 100% for Ck-18 with a mean value 348U/L with a highly significant difference in CHCV group in comparison to control group. This is in agreement with Gallo et al., [30].

Therefore, serum Ck-18 is more valuable than serum AFP for the diagnosis of HCC in contrast to Dom-Gene et al., [27] who said that Ck-18 is less valuable than AFP for the diagnosis of HCC because AFP is produced by the tumor, and only tumors originating in the gastrointestinal tract or yolk sac tumors complicated by hepatic metastases are likely to be mistaken clinically for HCC.

In conclusion, serum CK18 levels are significantly high in patients with CHCV; patients with HCC have the highest serum levels that might correlate its pathophysiology. Serum CK-18 levels may be useful for monitoring disease activity in CHCV, liver cirrhosis and HCC patients. The combination of AFP and CK18 increased the sensitivity of detection for HCC.

## Ethical Approval

All authors hereby declare that the study protocol has been examined and approved by the appropriate ethics committee and has therefore been performed in accordance with the ethical standards laid down in the 1964 Declaration of Helsinki.
